# Effects of Raman Labeling Compounds on the Stability and Surface-Enhanced Raman Spectroscopy Performance of Ag Nanoparticle-Embedded Silica Nanoparticles as Tagging Materials

**DOI:** 10.3390/bios14060272

**Published:** 2024-05-26

**Authors:** Cho-Hee Yang, Hye-Seong Cho, Yoon-Hee Kim, Kwanghee Yoo, Jaehong Lim, Eunil Hahm, Won Yeop Rho, Young Jun Kim, Bong-Hyun Jun

**Affiliations:** 1 Department of Bioscience and Biotechnology, Konkuk University, Seoul 05029, Republic of Koreajoh0302@konkuk.ac.kr (H.-S.C.);; 2 Nanophilia Inc., Gwacheon 13840, Republic of Korea; 3 School of International Engineering and Science, Jeonbuk National University, Jeonju 54896, Republic of Korea

**Keywords:** surface-enhanced Raman spectroscopy, Raman labeling compound, SERS tags, SiO_2_@Ag, Ag NPs, stability, period, pH, temperature

## Abstract

Surface-enhanced Raman spectroscopy (SERS) tagging using silica(SiO_2_)@Ag nanoparticles (NPs) is easy to handle and is being studied in various fields, including SERS imaging and immunoassays. This is primarily due to its structural advantages, characterized by high SERS activity. However, the Ag NPs introduced onto the SiO_2_ surface may undergo structural transformation owing to the Ostwald ripening phenomenon under various conditions. As a result, the consistency of the SERS signal decreases, reducing their usability as SERS substrates. Until recently, research has been actively conducted to improve the stability of single Ag NPs. However, research on SiO_2_@Ag NPs used as a SERS-tagging material is still lacking. In this study, we utilized a Raman labeling compound (RLC) to prevent the structural deformation of SiO_2_@Ag NPs under various conditions and proposed excellent SiO_2_@Ag@RLC-Pre NPs as a SERS-tagging material. Using various RLCs, we confirmed that 4-mercaptobenzoic acid (4-MBA) is the RLC that maintains the highest stability for 2 months. These results were also observed for the SiO_2_@Ag NPs, which were unstable under various pH and temperature conditions. We believe that SERS tags using SiO_2_@Ag NPs and 4-MBA can be utilized in various applications on based SERS because of the high stability and consistency of the resulting SERS signal.

## 1. Introduction

Surface-enhanced Raman spectroscopy (SERS) is an analytical technique that amplifies Raman scattering by adsorbing a substance onto a nanoscale metal surface. This enables the observation of the signal with high sensitivity, facilitating the detection of even very low substance concentrations [1,2,3,4,5]. This method, which harnesses the optical properties of metals, is driven by incident light on the surface of the material and the generation of a strong electric field between the metal and light. This results in collective oscillations or localized surface plasmon resonance (LSPR), resulting in very strong Raman intensities [4,6,7,8,9]. In addition, the bonding, orientation, and morphology of nanomaterials, which serve as SERS substrates for target molecules and their surfaces, can modify their physical or chemical properties, allowing for quantitative and qualitative analyses of even the smallest molecules [10,11].

SERS tags are constructed by incorporating a Raman labeling compound (RLC) as a specific organic Raman reporter. Usage or Utilization a SERS-tagging material labeled with RLC, which results in a unique spectrum for each material [5,12,13], can overcome the shortcomings of direct detection methods, where complex signal intensities make it difficult to precisely analyze Raman signals [12,14]. SERS tags can also be used as a substrate for biological targeting by adding biomolecular recognition elements such as antibodies [12,14,15,16,17], and RLCs with functional groups, such as amines or carboxyl groups, at the end can improve the dispersibility of nanoparticles (NPs) due to their charge. Owing to these advantages, a SERS-tagging material can be used for the targeting and imaging of small molecules, DNA, proteins, cells, tissues, and tumors in vivo [3,17,18,19,20,21,22,23,24,25,26]. However, because LSPR is sensitive to factors such as the shape, size, and concentration of the SERS substrate, it is very important to control it structurally and uniformly to increase signal consistency [1,9,10,13,27,28].

In a recent study, Ag NP-embedded silica NPs (SiO_2_@Ag NPs) were utilized as a SERS-tagging material owing to their structural features with many hotspots and the high plasmonic properties of Ag NPs [1,4,9,23,29,30,31]. The advantage of using the silica template is that it retains more hot spots due to the structural advantage. It has the advantage that the electromagnetic field can be amplified due to the increased number of hot spots, resulting in higher SERS activity. In addition, surface plasmon resonance can be converted into the NIR region. It allows the deep penetration of biological tissue with minimal absorption by the generated NIR light. Due to these advantages, it is widely used in hazardous substance detection, bioimaging, and therapeutic applications [32,33,34]. Although SiO_2_@Ag NPs offer the opportunity to exploit Ag NPs effectively and controllably, Ag NPs can become structurally unstable due to dissolution or Ostwald ripening during storage in the colloidal phase [35]. This unstable structure reduces the effectiveness of the hotspot and LSPR of SiO_2_@Ag@RLC NPs (as a SERS-tagging material), which can have a significant impact on the consistency of the SERS signal [32,36]. Several studies have been conducted to prevent the instability of Ag NPs [35,37,38,39,40]. However, to the best of our knowledge, there have been no studies on the stability of using an RLC when SiO_2_@Ag NPs are used as a SERS-tagging material. Therefore, economical and reliable research is required to improve the stability and consistency of the SERS signal when structurally affected SiO_2_@Ag NPs are used as a SERS-tagging material.

In this study, we aimed to stabilize the structure of SiO_2_@Ag NPs under various conditions (storage time, pH, and temperature). To achieve this, we proposed a method using 4-mercaptobenzoic acid (4-MBA), 4-aminothiolphenol (4-ATP), and 4-fluorobenzenethiol (4-FBT) as RLCs with different functional end groups. The method of introducing RLC immediately after the synthesis of SiO_2_@Ag NPs (SiO_2_@Ag@RLC-Pre NPs) not only stabilizes the structure but also improves the consistency of the SERS signal, thereby improving the suitability of SiO_2_@Ag NPs as a SERS-tagging material [41]. Among the different RLCs, we found that 4-MBA had the best effect on structural stability, even under harsh environmental conditions (2 months, pH 2, or a temperature of 55 °C), which also had a positive impact on the consistency of the SERS signal. We believe that our proposed method can be used in many studies based on the stability and high consistency of the SERS signal induced by the SiO_2_@Ag NPs as a SERS-tagging material.

## 2. Materials and Methods

### 2.1. Chemicals and Reagents

Ammonium hydroxide (NH_4_OH, 25–28%) was purchased from Daejung (Siheung, Republic of Korea). Ethanol (98%), tetraethyl orthosilicate (TEOS), 3-Mercaptopropyl trimethoxysilane (MPTS), poly(vinyl pyrrolidone) (PVP) (average molecular weight, ~40,000), silver nitrate (AgNO_3_, 99.99+%), ethylene glycol (EG), octylamine, 4-FBT, 4-ATP, and 4-MBA were purchased from Sigma-Aldrich (St. Louis, MO, USA) and used without further purification.

### 2.2. Preparation of SiO_2_@Ag NPs

TEOS was used as the precursor and NH_4_OH as the catalyst to prepare SiO_2_. Initially, 40 mL of ethanol and 4.5 mL of NH_4_OH were added to the round bottom (RB) flask and stirred at 500 rpm for 5 min in a water bath heated to 60 °C. Then, 1.6 mL of TEOS was added, and the reaction proceeded at 700 rpm for 1 h. After the reaction, the RB flask was immersed in a water bath and cooled at 700 rpm for 3 h at room temperature (25 °C). Finally, uniformly sized SiO_2_ NPs with a diameter of approximately 170 nm were produced and dispersed in ethanol as a colloidal phase.

To synthesize SiO_2_@Ag NPs, surface modification of the hydroxyl group (-OH) to a thiol group (-SH) on the surface of SiO_2_ was first carried out using MPTS. After mixing 0.5 mL of SiO_2_ (50 mg/mL) with 25 μL of MPTS and 5 μL of NH_4_OH and stirring overnight, the MPTS-treated SiO_2_ NPs were centrifuged at 15,000 rpm for 10 min. Subsequently, they were washed with ethanol and dispersed.

To form Ag shells on MPTS-treated SiO_2_ NPs, 25 mL of PVP (5 mg) dissolved in EG, 0.2 mL of MPTS-SiO_2_ NPs (50 mg/mL), 25 mL of AgNO_3_ (26 mg) in EG, and 41.4 μL of octylamine as a reducing agent were added in order and mixed. The resulting mixture was stirred at 700 rpm for 30 min, and the resulting SiO_2_@Ag NPs were washed and dispersed in ethanol several times to maintain them in the colloidal phase.

### 2.3. Synthesis of Various Types of RLCs

Three types of RLCs were used for the SiO_2_@Ag NPs: 4-ATP, 4-MBA, and 4-FBT. The ratio of SiO_2_@Ag to RLC was 1:1. The concentration of the RLCs was 0.3 mM for the duration test and 0.1 mM for the pH and temperature tests. Each RLC was prepared by dispersion in the ethanol phase, and the mixture was stirred for 1 h. Subsequently, it was washed and dispersed several times with ethanol to obtain a final particle concentration of 1 mg/mL.

### 2.4. Reaction under pH Conditions

The pH was adjusted to 2 (acidic), 7 (neutral), and 11 (basic), and the buffers were prepared by adjusting the amount of ammonium acetate and HNO_3_ (the ratio of SiO_2_@Ag NPs to the pH buffer was 1:1). Then, 0.2 mL of SiO_2_@Ag NPs (1 mg/mL) and 0.2 mL of pH buffer were mixed and stirred for 3 days. The finished mixture was washed twice with distilled water and three times with ethanol to a final concentration of 1 mg/mL.

### 2.5. Reaction under Temperature Conditions

In the case of temperature, we used 4 °C (refrigeration), 25 °C (room temperature), and 55 °C (high temperature). Considering that the vaporization temperature of ethanol is 78–80 °C, 55 °C was set as the high temperature. Then, 0.2 mL of SiO_2_@Ag and 0.2 mL of ethanol were mixed in a 1:1 ratio and stored under the same conditions for 3 days at each temperature. The final mixture was washed several times with ethanol and dispersed to a final concentration of 1 mg/mL.

### 2.6. Instruments and Measurement 

The transmission electron microscopy (TEM) images were obtained using a JEOL (JEM-1010/JEOL/JP) instrument operating at 80 kV. Before measurement, 20 μL of NPs dispersed in the colloidal phase were dropped onto a copper (Cu) grid and allowed to dry completely. 

UV-Vis spectra were measured with a Ratio Beam Spectrophotometer U-5100 (Hita-chi) using a polystyrene cuvette with a wavelength of 10 mm and a size of 12.5 × 12.5 × 45 mm. The dilution factor was 1/10 for all samples in the 290–1100 nm range. The values of all measured UV-Vis spectra were normalized to 430 nm. The sample concentration was determined to be 1 mg/mL.

For SERS measurements, a DXR Raman Microscope (Thermo Fisher Scientific, Waltham, MA, USA) was utilized, which outputs SERS spectra in the approximate 500–2000 cm^−1^ range using a 532-nm light laser. The sample concentration was determined to be 1 mg/mL.

The laser intensity was 10 mW, and eight measurements of 2 s each were taken using a lens at 10× magnification for a total exposure time of 16 s. The size of the laser beam spot was approximately 2.0 μm. The samples were measured using a capillary tube made of soda-lime glass, filled with colloidal NPs, and fixed on a glass slide (Figure S1). All data are presented as mean values obtained from triplicate measurements.

## 3. Results and Discussion

### 3.1. Characteristics of SiO_2_@Ag NPs

**Figure 1:**
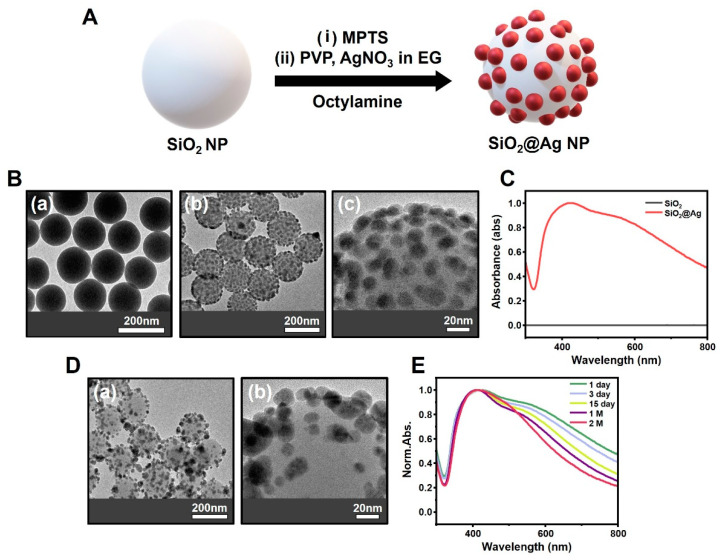
(**A**) Synthesis of SiO_2_@Ag nanoparticles (NPs). (**B**) Transmission electron microscopy (TEM) images of (**a**) SiO_2_ NPs and (**b**) SiO_2_@Ag NPs, and (**c**) the surface structure of SiO_2_@Ag NPs. (**C**) Comparison of UV-Vis spectra between SiO_2_ NPs and SiO_2_@Ag NPs. (**D**) TEM images of structural changes in (**a**) SiO_2_@Ag NPs and (**b**) the surface structure after 2 months depending on the storage period. (**E**) UV-Vis spectra of SiO_2_@Ag NPs from 1 day to 2 months.

The SiO_2_@Ag NPs were synthesized using a previously reported method (Figure 1A) [24,42]. First, SiO_2_ NPs were synthesized via the Stöber method, which is a preparation method based on the sol-gel technique that is relatively simple and inexpensive and yields particles with a uniform size and shape, which is useful for mass production [29,43]. The synthesized SiO_2_ NPs formed uniformly shaped spheres with a diameter of approximately 170 nm (Figure 1(Ba)). The spherical SiO_2_ NPs formed are not only more stable and highly dispersible compared to other forms but also have a large surface area, which can increase the catalytically active portion and thus increase the efficiency of the reaction [31,44,45].

SiO_2_@Ag NPs were synthesized by the surface modification of many hydroxyl groups (-OH) present in SiO_2_ NPs into thiol groups (-SH) using MPTS and then adding AgNO_3_ as a precursor and octylamine as a reducing agent (Figure 1A) [27,46]. TEM images and UV-Vis spectra were used to identify the synthesized SiO_2_@Ag NPs. From the TEM images, the SiO_2_@Ag synthesized was approximately 200 nm in size (Figure 1(Bb,c)). Comparing the absorbance of single SiO_2_ NPs and SiO_2_@Ag NPs by UV-Vis spectroscopy, the wavelength at the peak absorption of SiO_2_@Ag NPs was found to be high at approximately 430 nm (Figure 1C) [47]. This result has been shown to contribute to the characteristic LSPR band of Ag NPs alone [1,4,44], showing that the incorporation of Ag NPs into silica was well achieved.

### 3.2. Comparison of Particle Changes over Time for SiO_2_@Ag NPs

SiO_2_@Ag NPs are structurally characterized as having an excellent LSPR, and the addition of RLCs gives them a great advantage in studies using a SERS-tagging material [36,41]. However, because of the nature of Ag NPs, dissolution or Ostwald ripening occurs during storage in the colloidal phase, causing structural deformation and poor signal consistency [9,35]. The synthesized SiO_2_@Ag NPs were stored in ethanol at approximately 4 °C in refrigeration. To observe the structural changes in SiO_2_@Ag NPs during the storage period, we compared the TEM images and UV-Vis spectra of SiO_2_@Ag NPs at 1 day and 2 months after synthesis. The TEM images showed that the structure of the SiO_2_@Ag NPs was significantly disrupted in the particles after 2 months compared to the particles after 1 day of synthesis, owing to the aggregation and desorption of Ag NPs from the SiO_2_ surface (Figure 1D). To confirm the stability of the structure more precisely, UV-Vis spectra, accompanied by quantitative analysis, showed a proportional decrease in absorbance at long wavelengths (approximately 600–800 nm) over time (Figure 1E). This occurs due to interparticle plasmonic oscillation coupling depending on the gap of the plasmonic nanoparticle, resulting in a shift of the LSPR band including the dipole mode of the nanoparticle, which is a proximity effect. The shift of the LSPR band is redshift as the gap of the plasmonic nanoparticle becomes smaller, increasing the absorbance of long wavelengths. Ag NPs introduced into SiO_2_ NPs by the proximity effect are redshifted due to a small gap structure on the 1 day, increasing the absorbance of the long wavelength, and at 2 M, structural deformation occurs due to the low stability of Ag NPs, increasing the size of the gap and reducing the absorbance at the long wavelength compared to the 1st day [48]. Additionally, this shows that despite the use of PVP to prevent the dispersion and aggregation of Ag NPs during the synthesis process [45,46,49,50,51], the overall structural modification of the SiO_2_@Ag NPs was due to the unstable nature of the Ag NPs, which re-emerged over time.

**Figure 2:**
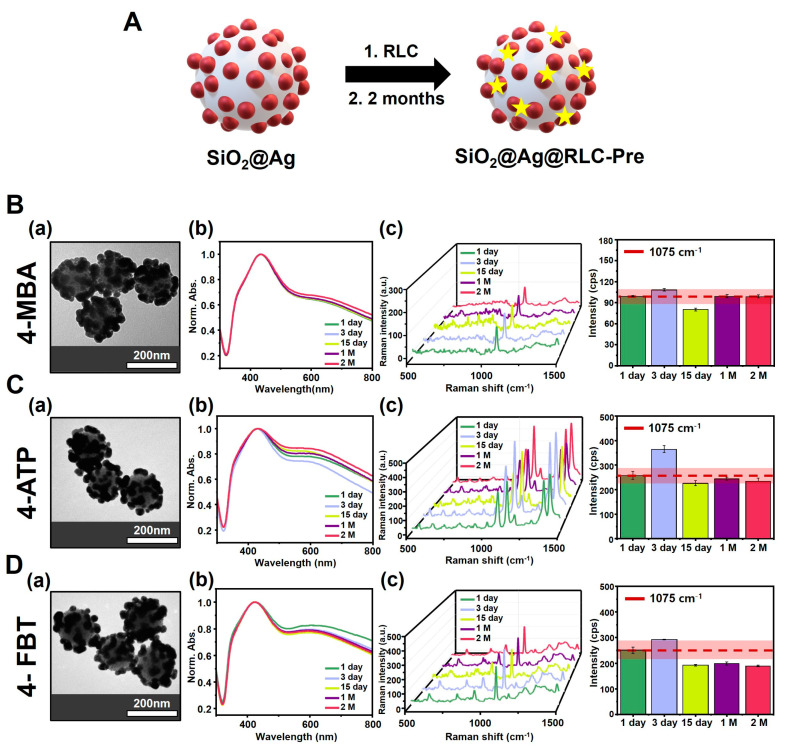
(**A**) Schematic diagram of SiO_2_@Ag@ Raman labeling compound (RLC)-Pre NPs with different types of RLCs: 4-aminothiophenol (4-ATP), 4-fluorothiophenol (4-FBT), and 4-mercaptobenzoic acid (4-MBA), introduced immediately after synthesis. TEM images and UV-Vis spectra of SiO_2_@Ag@RLC-Pre NPs with **B**(**a**,**b**) 4-MBA, **C**(**a**,**b**) 4-ATP, and **D**(**a**,**b**) 4-FBT, which maintained a stable structure over time. The 3D Surface-enhanced Raman spectroscopy (SERS) spectra and Raman signal intensity of SiO_2_@Ag@RLC-Pre NPs showing a consistent and stable SERS signal over time, achieved by the introduction of the RLCs **B**(**c**) 4-MBA, **C**(**c**) 4-ATP, and **D**(**c**) 4-FBT. The spectra were recorded at 1075 cm^−1^. The red line is the intensity value of 1 day to indicate the degree of change in Raman signal consistency. The surrounding red area indicates the intensity of variation of ±10%.

Therefore, we sought to evaluate the structural stability of SiO_2_@Ag@RLC NPs and their impact on the consistency of the SERS signal by introducing RLCs into the SiO_2_@Ag NPs for use as a SERS-tagging material. To confirm the stability of SiO_2_@Ag NPs in the presence of RLCs, we compared the differences between two groups of SiO_2_@Ag NPs. One group (SiO_2_@Ag@RLC-Post) (Figure S2A) was the SiO_2_@Ag NPs, in which RLCs were introduced after a certain incubation time. The other group (SiO_2_@Ag@RLC-Pre) (Figure 2A) consisted of SiO_2_@Ag NPs in which RLCs were introduced immediately after the synthesis of SiO_2_@Ag NPs. The RLCs used were 4-ATP, 4-FBT, and 4-MBA, which contain thiol groups (-SH) with a strong affinity for Ag NPs [23]. The time period was divided into five intervals (1 day, 3 days, 15 days, 1 month, and 2 months), and the TEM images, UV-Vis spectra, and SERS spectra of the SiO_2_@Ag NPs were measured. For SiO_2_@Ag@RLC-Post NPs, the TEM images showed significant structural deformation, similar to the results for SiO_2_@Ag NPs after 2 months (Figure S2(Ba,Ca,Da)) In addition, the UV-Vis spectra showed a significant change in absorbance at long wavelengths over time, in the order of 30–40% (Figure S2(Bb,Cb,Db)). These results suggest that introducing RLCs into SiO_2_@Ag NPs that have already undergone structural modification over time is not effective for structural prophylaxis. In contrast, the TEM images of SiO_2_@Ag@RLC-Pre NPs showed a more structurally stable appearance compared to the control, even after 2 months (Figure 2(Ba,Ca,Da). UV-Vis spectra also showed a smaller change in absorbance at long wavelengths compared to SiO_2_@Ag@RLC-Post NPs, with a change of less than 10% (Figure 2(Bb,Cb,Db)). The structurally stabilized SiO_2_@Ag@RLC-Pre-NPs also positively affected the consistency of the SERS signal. SERS spectra was observed in the range 500–1500 cm^−1^, which is an appropriate range to measure the stability of signal changes, based on the Raman spectrum results for the entire range (200–3100 cm^−1^) (Figure S3). In the SERS spectra, the consistency of the SERS signal for each RLC was compared based on the aromatic C-H stretching peak of 4-ATP, 4-FBT, and 4-MBA at 1075 cm^−1^. Similar to previous results, SiO_2_@Ag@RLC-Post NPs showed low consistency of the SERS signal with about 30–45% variation in all RLCs (Figure S2(Bc,Cc,Dc)). These results suggest that structural modifications over time significantly affect the consistency of the SERS signal. However, for SiO_2_@Ag@RLC-Pre NPs, the Raman intensity was similar to that of the control (1 day) and showed a high consistency of the SERS signal for all RLCs: 4-MBA (7.27%), 4-ATP (16.87%), and 4-FBT (21.24%) (Figure 2(Bc,Cc,Dc)). This confirms that the RLCs introduced immediately after synthesis stabilized the structure of the SiO_2_@Ag NPs by preventing the time-dependent instability of the Ag NPs. Among the different RLCs used, the introduction of 4-MBA resulted in the narrowest variation in the UV-Vis spectra and the best consistency of the SERS signal.

### 3.3. Reaction of SiO_2_@Ag NPs under pH Conditions

SiO_2_@Ag NPs can be used for pH detection in various fields, such as environmental, medical, and industrial applications; however, Ag NPs are sensitive to H^+^ ion concentrations and can be easily modified [44,49,52]. This means that the physical and chemical properties of the surface of SiO_2_@Ag NPs can be changed when exposed to different pH conditions, and these changes in response to different environmental conditions can be a disadvantage for powerful SERS substrates for detecting specific substances [40,53].

To determine the effect of pH, we confirmed the structural changes in the SiO_2_@Ag NPs using TEM images and UV-Vis spectra at pH 2 (acidic), pH 7 (neutral), and pH 11 (basic) (Figure S4). The TEM images confirmed that Ag NPs on the surface of SiO_2_ NPs were aggregated and desorbed under conditions of pH 2 and 7 compared to control NPs (pH 7 in Ethanol) (Figure S4A). In particular, most structural changes occurred at pH 2, which is a very low pH condition. The stability of the particles was confirmed by UV-Vis spectra, which showed a changing structure due to the aggregation and desorption of Ag NPs as the pH decreased. This was indicated by the difference in absorbance at longer wavelengths compared to the control NPs (Figure S4B). The structural change depending on pH is because as pH decreases, the acidity of Ag NPs increases and more H^+^ ions are generated. Therefore, the negative surface charge of the Ag NPs was canceled out, causing a loss in charge and a reduction in electrostatic stabilization, ultimately leading to agglomeration of the Ag NPs. This was accompanied by the desorption of Ag NPs from the SiO_2_ surface, resulting in low absorbance at long wavelengths. In contrast, at pH 11, which is rich in OH^−^ ions, we observed little structural change because no ion exchange with the Ag NPs occurred [52].

**Figure 3:**
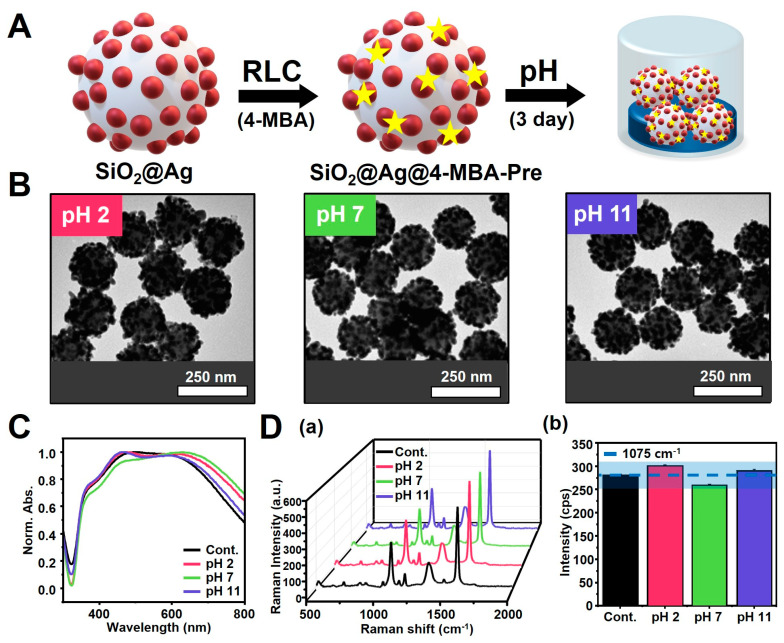
Effect of SiO_2_@Ag@4-MBA-Pre NPs with RLC(4-MBA) introduced immediately after synthesis of SiO_2_@Ag NPs, showing the highly stable structure and consistency of SERS signal under different pH conditions. (**A**) Schematic diagram of SiO_2_@Ag@4-MBA-Pre NPs. (**B**) TEM images and (**C**) UV-Vis spectra of SiO_2_@Ag@4-MBA-Pre NPs showing similar structures at different pH levels. (**D**) Consistency of SERS signals with pH of SiO_2_@Ag@4-MBA-Pre NPs: (**a**) 3D SERS spectra and (**b**) Raman signal intensity, with similar values, recorded at 1075 cm^−1^. The blue line is the intensity value of 1 day to indicate the degree of change in consistency of the SERS signal. The surrounding blue area indicates the intensity of variation of ±10%.

However, for SiO_2_@Ag NPs to be used as a SERS-tagging material, a structure that is insensitive to various environmental conditions is required. To obtain SiO_2_@Ag NPs that maintain a constant structure under different pH environments, we synthesized SiO_2_@Ag@RLC NPs treated with RLCs. The RLC 4-MBA had the most beneficial effect on structural stability over time. The reactions proceeded under different pH conditions for 3 days. TEM images showed that SiO_2_@Ag@4-MBA-Pre NPs (Figure 3A), in which 4-MBA was introduced immediately after synthesis, had an almost similar structure at all pH conditions (Figure 3B) compared to the TEM images (Figure S5B) of SiO_2_@Ag@4-MBA-Post NPs (Figure S5A), in which the structure of SiO_2_@Ag NPs had already changed owing to the pH.

Likewise for the UV-Vis spectra, the variation in absorbance for SiO_2_@Ag@4-MBA-Pre NPs was very narrow and similar at all pH levels (Figure 3C), unlike for SiO_2_@Ag@4-MBA-Post NPs (v5C), where the absorbance varied significantly with pH. These results indicated that the introduction of 4-MBA into SiO_2_@Ag NPs led to high structural stability under different pH conditions.

Finally, differences in structural stability were also revealed in the SERS measurements. SiO_2_@Ag@4-MBA-Post NPs showed a consistency of the SERS signal outside ±10% at the levels of pH 2, pH 7, and pH 11 based on the Raman intensity of the control (Figure S5(Da,b)). The difference in intensity was highly dependent on the pH conditions. Signal decreases of 25.56%, 58.15%, and 61.29% were observed at pH 2, 7, and 11, respectively. These results imply that the exposure of SiO_2_@Ag NPs to different pH conditions, even if they are structurally similar, as in the case of pH 11, significantly changed the environment in which they were stored (in ethanol), affecting their optical properties and the consistency of the SERS signal. On the other hand, the SiO_2_@Ag@4-MBA-Pre NPs showed higher consistency in the SERS signal, varying within ±10% at all pH conditions (Figure 3(Da,b)). Thus, all results under different pH conditions showed that 4-MBA enhanced the stability of the structure of SiO_2_@Ag NPs under acidic, neutral, and basic conditions, resulting in particles with high utilization as SERS substrates.

### 3.4. Reaction of SiO_2_@Ag NPs under Temperature Conditions

Ag NPs are also sensitive to temperature-induced structural changes owing to aggregation. In particular, the size of Ag NPs gradually increases with increasing temperature, creating an environment in which interparticle aggregation can occur easily [54,55,56,57]. Therefore, we aimed to reduce the highly temperature-sensitive nature of Ag NPs and increase their utility as a SERS-tagging material by using SiO_2_@Ag NPs that maintain a stable structure at various temperatures. 

To confirm the temperature effect on SiO_2_@Ag NPs, we conducted the tests under three temperature conditions: 4 °C (refrigeration), 25 °C (room temperature), and 55 °C (high temperature); the other conditions were the same as those for the method used in pH for accurate comparison. Figure S6 shows the structural changes in the SiO_2_@Ag NPs under three different temperature conditions. As shown in the TEM images, at 4 °C and 25 °C, little deformation was observed for the structure of the synthesized SiO_2_@Ag NPs (Figure S6A). However, when treated at a high temperature of 55 °C, the SiO_2_@Ag NPs were not well dispersed in a stable state. The structure was greatly deformed and existed in an unstable state. These results indicate that Ag NPs become more sensitive and have an increased aggregation tendency with increasing temperature [54]; thus, the Ag NPs that were evenly dispersed on the SiO_2_ surface reacted with each other. As a result, the Ag NPs on the SiO_2_ surface aggregated and desorbed, resulting in structural modification of the SiO_2_@Ag NPs. From the UV-Vis spectra, the highest absorption peak was observed at a long wavelength in the case of high temperature (55 °C), and a change in absorbance was observed compared to control NPs (4 °C in refrigeration) (Figure S6B). The reason for this may be due to the fact that when structural transformation occurred, aggregation between single Ag NPs and SiO_2_@Ag NPs increased the overall diameter of the particles being measured.

**Figure 4:**
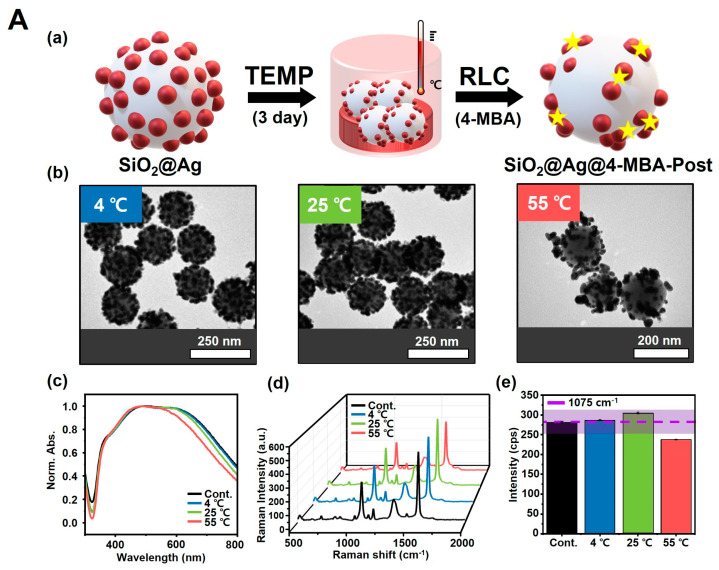

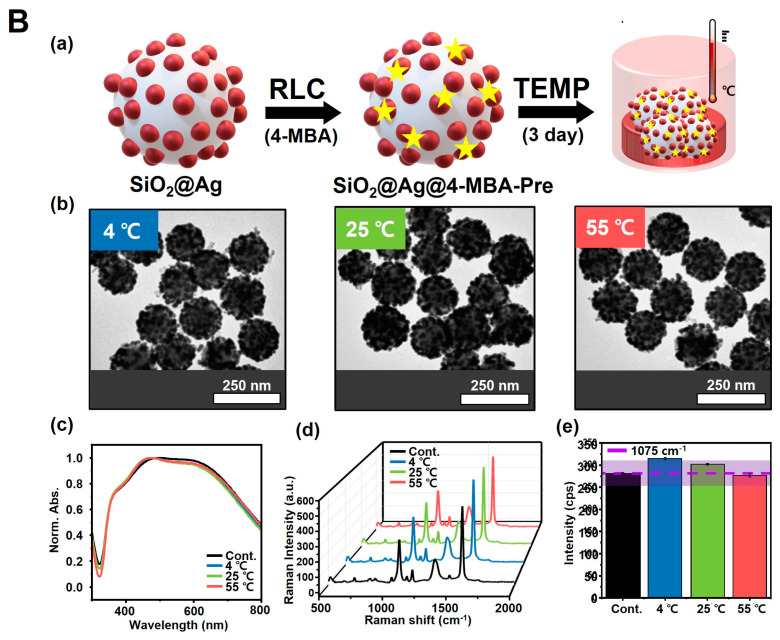
Effect of introducing an RLC (4-MBA) into SiO_2_@Ag NPs, showing structural modifications under various temperature conditions. (**A**) SiO_2_@Ag@4-MBA-Post NPs. Results of introducing 4-MBA into SiO_2_@Ag NPs whose structure was modified by different temperatures (TEMP): 4 °C, 25 °C, and 55 °C. (**a**) Schematic diagram of SiO_2_@Ag@4-MBA-Post NPs. (**b**) TEM images showing the most deformed particle structure at a high temperature (55 °C). (**c**) UV-Vis spectra showing the change in absorbance of SiO_2_@Ag NPs whose structural modification was induced by temperature. Consistency of the SERS signal of SiO_2_@Ag@4-MBA-Post NPs based on the different temperatures: (**d**) 3D SERS spectra and (**e**) Raman signal intensity, recorded at 1075 cm^−1^, which are outside the stable signal range at a high temperature. The purple line is the intensity value of 1 day to indicate the degree of change in consistency of the SERS signal. The surrounding purple area indicates the intensity of variation of ±10%. (**B**) Results of stabilized SiO_2_@Ag@4-MBA-Pre NPs without structural changes at 4 °C, 25 °C, and 55 °C. (**a**) Schematic diagram of SiO_2_@Ag@4-MBA-Pre NPs. (**b**) TEM images and (**c**) UV-Vis spectra of SiO_2_@Ag@4-MBA-Pre NPs showing similar structures at different temperatures. Consistency of the SERS signal of SiO_2_@Ag@4-MBA-Pre NPs based on the different temperatures: (**d**) 3D SERS spectra and (**e**) Raman signal intensity, recorded at 1075 cm^−1^, which are in a stable range at all temperatures.

Therefore, we sought to determine whether the SiO_2_@Ag NPs could remain stable under different temperature conditions owing to the introduction of an RLC (4-MBA) without any structural modifications caused by the instability of the Ag NPs. As a result, SiO_2_@Ag@4-MBA-Post NPs (Figure 4(Aa)) showed a significant structure disruption at a high temperature in the TEM images (Figure 4(Ab)), while SiO_2_@Ag@4-MBA-Pre NPs (Figure 4(Ba)) showed a well-dispersed structure with little change in structure at all temperature conditions (4 °C, 25 °C, and 55 °C) (Figure 4(Bb)). Furthermore, as shown in the UV-Vis spectra and SERS spectra, in contrast to SiO_2_@Ag@4-MBA-Post NPs (Figure 4(Ac–e)), SiO_2_@Ag@4-MBA-Pre NPs exhibited much less variation in absorbance (Figure 4(Bc)) and high consistency of the SERS signal (Figure 4(Bd,e)), which varied within ±10% at all temperatures based on the Raman intensity of the control. These results indicate that the structure of the SiO_2_@ Ag NPs remained stable at high temperatures, indicating that 4-MBA has a stabilizing effect on the SiO_2_@Ag NPs under different temperature conditions. 

## 4. Conclusions

We compared the stability of SiO_2_@Ag@RLC NPs, which are widely used a SERS-tagging material, with respect to storage time, pH, and temperature. The TEM images, UV-Vis spectra, and SERS spectra showed that the structure of the SiO_2_@Ag NPs changed significantly over time. This was a result of the instability of the Ag NPs owing to Ostwald ripening during storage. However, the introduction of an RLC resulted in a stable metallic nanostructured surface and a reproducible SERS signal, even after 2 months, due to narrower changes in the UV-Vis spectra and SERS spectra, as well as Raman intensity values varying within ±10%. Moreover, among the three types of RLCs used, 4-MBA had the most significant effect on the stability of the structure of SiO_2_@Ag NPs, and the structure remained highly stable under harsh conditions, including high temperature (55 °C) and acidic (pH 2) conditions. Therefore, we believe that SiO_2_@Ag@RLC-Pre NPs, with RLCs introduced into SiO_2_@Ag NPs exhibiting strong Raman signals, can be used as an efficient SERS-tagging material with high versatility and low cost, exhibiting a stable structure and reliable SERS signal consistency under various conditions.

In experiments by pH and temperature, only one RLC (4-MBA) was used to perform all the stability experiments under various conditions (storage time, pH, and temperature) because 4-MBA was selected as the most effective among the various RLCs for a long period of time. Also, the SiO_2_@Ag NPs used in the experiments were performed using silica with a diameter of only 170 nm. These may be a limitation of this paper. However, because our study specifically evaluated the stability of SERS materials and Raman signals in the field of SERS-tagging under various conditions, this can be a valid evaluation standard compared to previous studies. Based on these results, we believe that this probe will be very promising as a SERS-tagging material.

## Data Availability

Data are contained within the article or Supplementary Materials.

## Supplementary Materials

The following supporting information can be downloaded at: https://www.mdpi.com/article/10.3390/bios14060272/s1, Figure S1. Schematic design of geometry Raman spectroscopy measured in solution; Figure S2. (A) Schematic diagram of SiO_2_@Ag@RLC-Post NPs with different type of RLCs (4-Aminothiophenol(4-ATP), 4-Fluorothiophenol(4-FBT), 4-Mercaptobenzoic acid(4-MBA)) introduced into SiO_2_@Ag NPs whose structure was modified after 2 months. TEM images and UV-Vis spectra of SiO_2_@Ag@RLC-Post NPs with B(a,b) 4-MBA, C(a,b) 4-ATP and D(a,b) 4-FBT showing structural aggregation and desorption occurring over time. The 3D SERS spectra and Raman signal intensity of SiO_2_@Ag@RLC-Post NPs show low consistency of SERS signal due to decreased structural stability over time despite the introduction of RLC with B(c) 4-MBA, C(c) 4-ATP and D(c) 4-FBT recorded at 1075 cm^−1^. (The red line is the intensity value of 1 day to indicate the degree of change in Raman signal reproducibility. And the surrounding red area indicates the intensity of variation of ±10%.); Figure S3. The 3D SERS spectrum measured in the entire range (200–3100 cm^−1^) for each RLC (4-MBA, 4-ATP and 4-FBT); Figure S4. (A) TEM images showing structural change of SiO_2_@Ag NPs depending on pH, unlike SiO_2_@Ag NPs (Cont.) stored in ethanol. (B) UV-Vis spectra of SiO_2_@Ag NPs treated under each pH condition (pH 2, pH 7, pH 11) for 3 days; Figure S5. Effect of SiO_2_@Ag@4-MBA-Post NPs with RLC (4-MBA) introduced on SiO_2_@Ag NPs whose structure is modified under different pH conditions. (A) Schematic diagram of SiO_2_@Ag@4-MBA-Post NPs. (B) TEM images of SiO_2_@Ag@4-MBA-Post NPs with different structural modifications at pH 2, 7, and 11. (C) UV-Vis spectra showing the change in absorbance of SiO_2_@Ag@4-MBA-Post NPs whose structural modification was induced by pH. (D) Consistency of SERS signal depending on the pH for SiO_2_@Ag@4-MBA-Post NPs: (a) 3D SERS spectra and (b) Raman signal intensity, recorded at 1075 cm^−1^, which are outside the stable signal range as the pH changes. The blue line is the intensity value of 1 day to indicate the degree of change in consistency of the SERS signal. The surrounding blue area indicates the intensity of variation of ±10%; Figure S6. (A) TEM images showing structural change of SiO_2_@Ag NPs depending on temperature, unlike SiO_2_@Ag NPs (Cont.) stored in ethanol. (B) UV-Vis spectra of SiO_2_@Ag NPs treated under each temperature condition (4 °C, 25 °C, 55 °C) for 3 days.
