# Supplementary material for: Effects of Raman Labeling Compounds on the Stability and Surface-Enhanced Raman Spectroscopy Performance of Ag Nanoparticle-Embedded Silica Nanoparticles as Tagging Materials
Source: Biosensors (Basel). 2024 May 26;14(6):272. doi: 10.3390/bios14060272 (PMC11201858; doi:10.3390/bios14060272)
Supplement: Supplementary file 1 [file biosensors-14-00272-s001.zip › biosensors-2995208-supplementary.pdf]

Supplementary Information

Effect of Raman Labeling Compounds on the stability and SERS performance of Ag NP-embedded silica NPs as tagging materials

Cho-Hee Yang†, Hye-Seong Cho†, Yoon-Hee Kim, Kwanghee Yoo, Jaehong Lim, Eunil Hahm, Won Yeop Rho, Young Jun Kim and Bong-Hyun Jun*

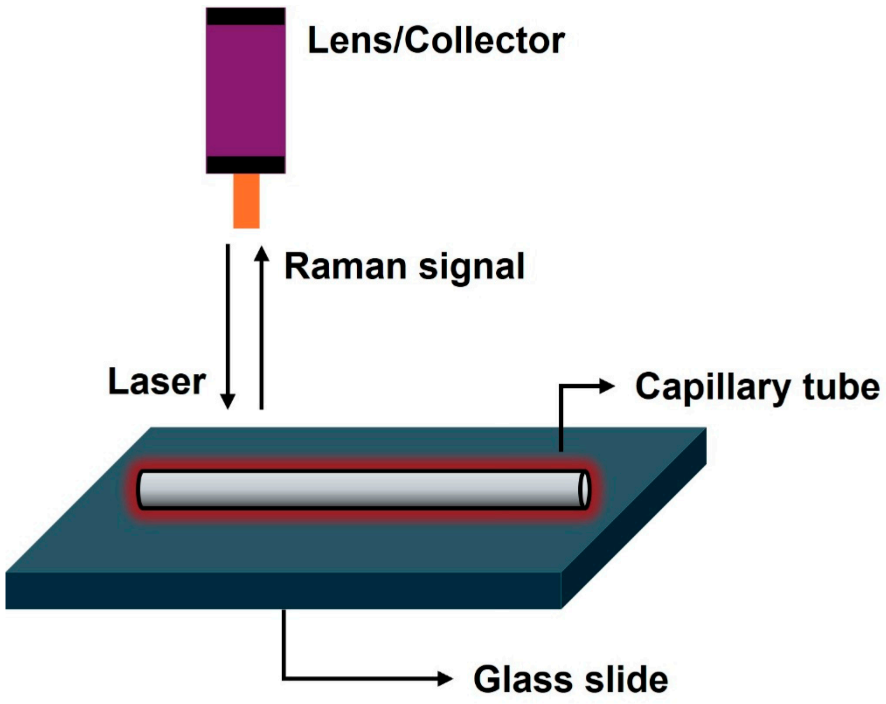

Figure S1. Schematic design of geometry Raman spectroscopy measured in solution.

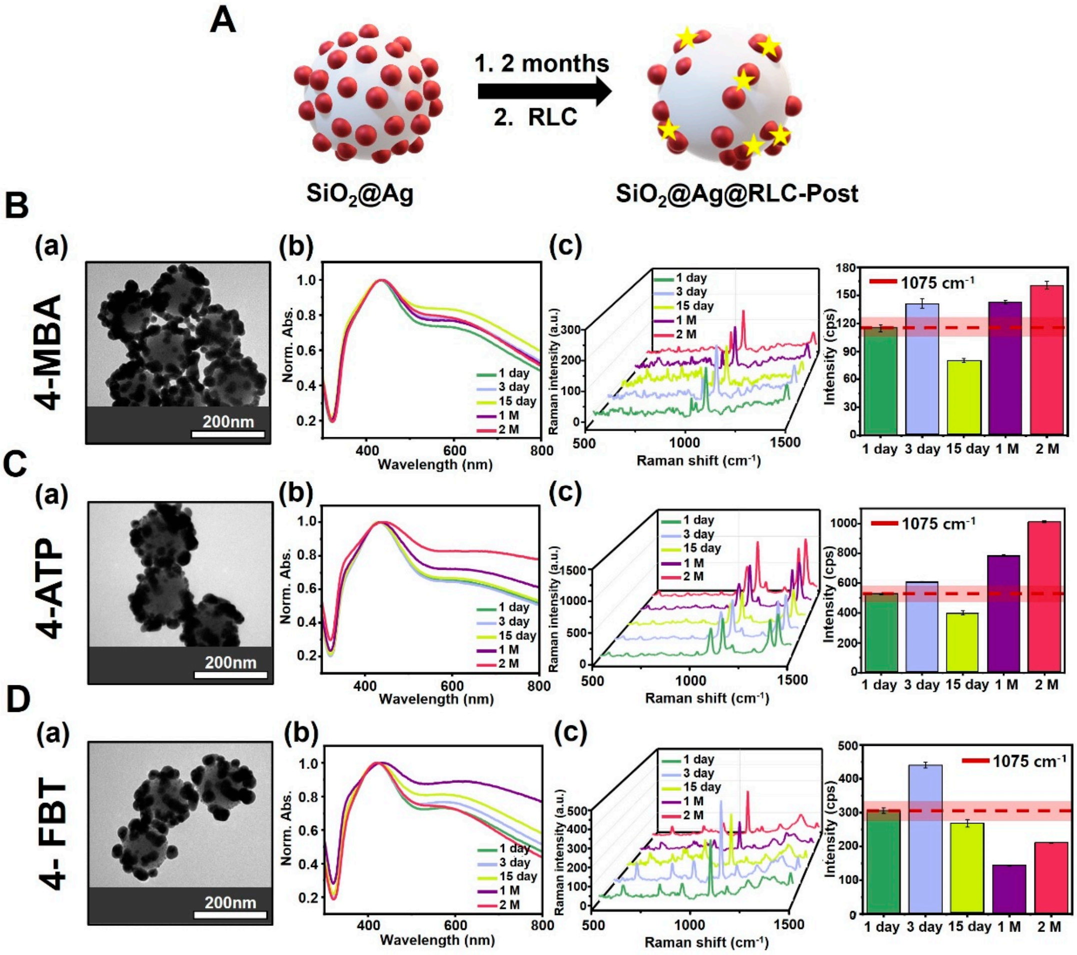

Figure S2. (A) Schematic diagram of SiO_2@Ag@RLC-Post NPs with different type of RLCs (4-Aminothiophenol(4-ATP), 4-Fluorothiophenol(4-FBT), 4-Mercaptobenzoic acid(4-MBA)) introduced into SiO_2@Ag NPs whose structure was modified after 2 months. TEM images and UV-Vis spectra of SiO_2@Ag@RLC-Post NPs with **B(a,b)** 4-MBA, **C(a,b)** 4-ATP and **D(a,b)** 4-FBT showing structural aggregation and desorption occurring over time. 3D SERS spectra and Raman signal intensity of SiO_2@Ag@RLC-Post NPs show low consistency of SERS signal due to decreased structural stability over time despite the introduction of RLC with **B(c)** 4-MBA, **C(c)** 4-ATP and **D(c)** 4-FBT recorded at 1075 cm^{-1}. (The red line is the intensity value of 1 day to indicate the degree of change in Raman signal reproducibility. And the surrounding red area indicates the intensity of variation of ±10%.)

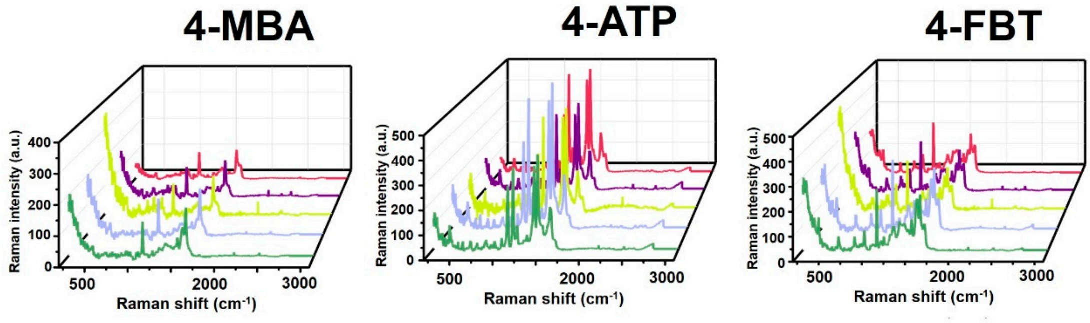

Figure S3. 3D SERS spectrum measured in the entire range (200-3100 cm^{-1}) for each RLC (4-MBA, 4-ATP and 4-FBT)

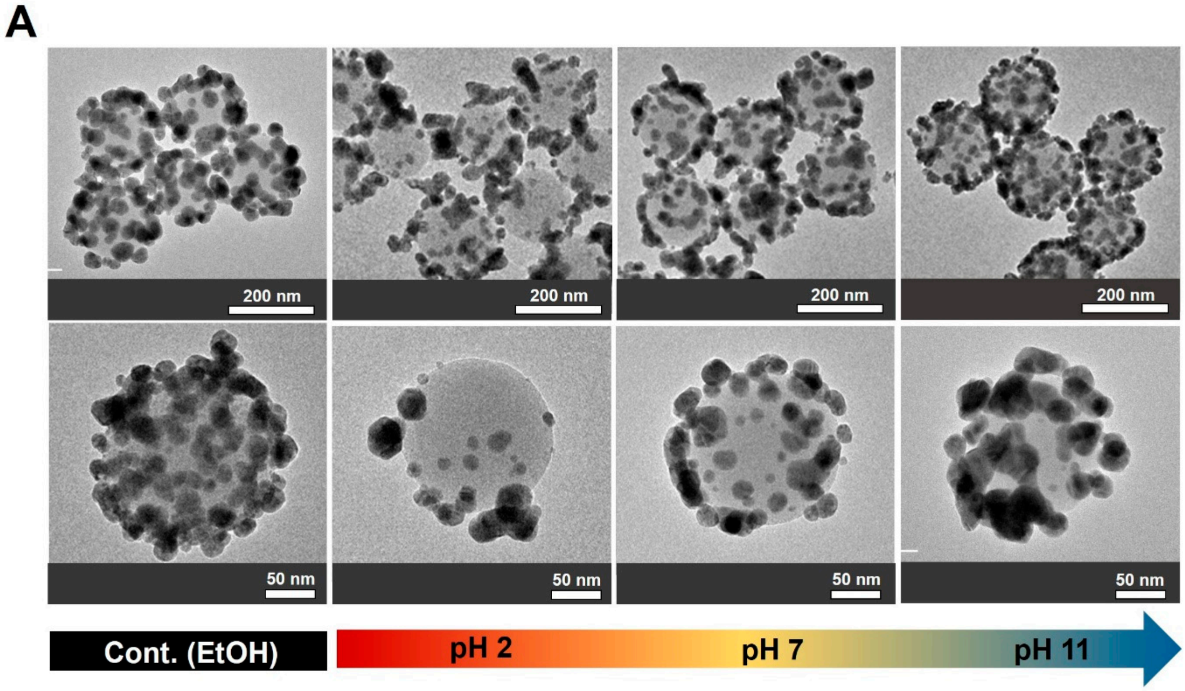

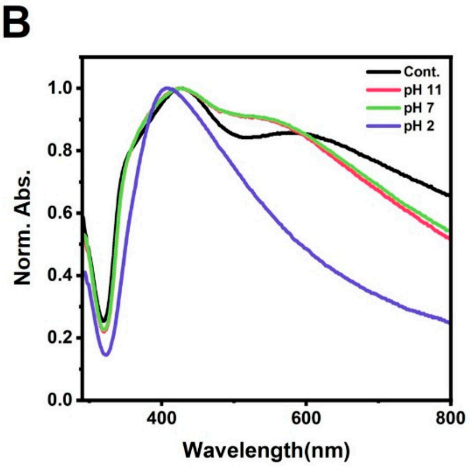

Figure S4. (A) TEM images showing structural change of SiO_2@Ag NPs depending on pH, unlike SiO_2@Ag NPs (Cont.) stored in ethanol. **(B)** UV-Vis spectra of SiO_2@Ag NPs treated under each pH condition (pH 2, pH 7, pH 11) for 3 days.

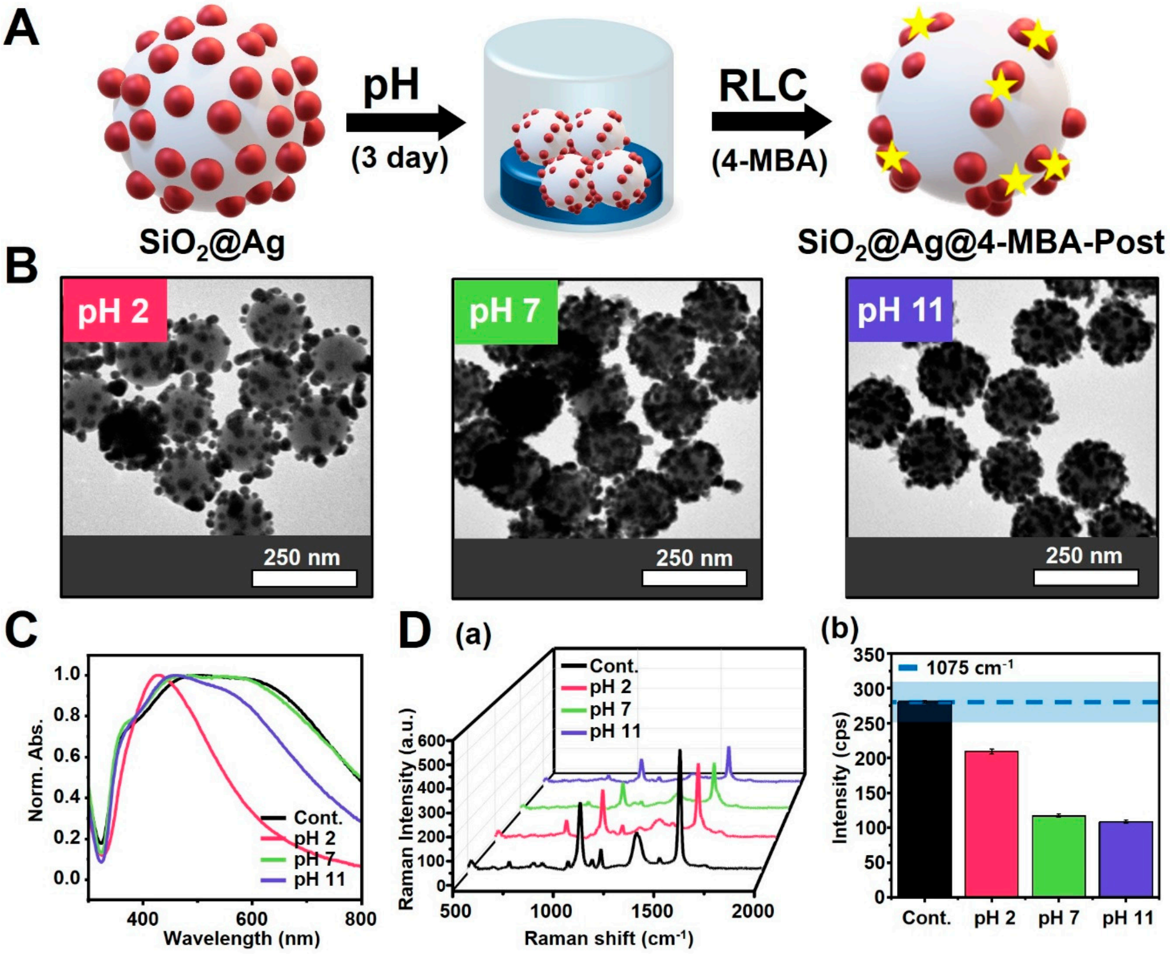

Figure S5. Effect of SiO_2@Ag@4-MBA-Post NPs with RLC (4-MBA) introduced on SiO_2@Ag NPs whose structure is modified under different pH conditions. **(A)** Schematic diagram of SiO_2@Ag@4-MBA-Post NPs. **(B)** TEM images of SiO_2@Ag@4-MBA-Post NPs with different structural modifications at pH 2, 7, and 11. **(C)** UV-Vis spectra showing the change in absorbance of SiO_2@Ag@4-MBA-Post NPs whose structural modification was induced by pH. **(D)** Consistency of SERS signal depending on the pH for SiO_2@Ag@4-MBA-Post NPs: **(a)** 3D SERS spectra and **(b)** Raman signal intensity, recorded at 1075 cm^{-1}, which are outside the stable signal range as the pH changes. The blue line is the intensity value of 1 day to indicate the degree of change in consistency of the SERS signal. The surrounding blue area indicates the intensity of variation of ±10%.

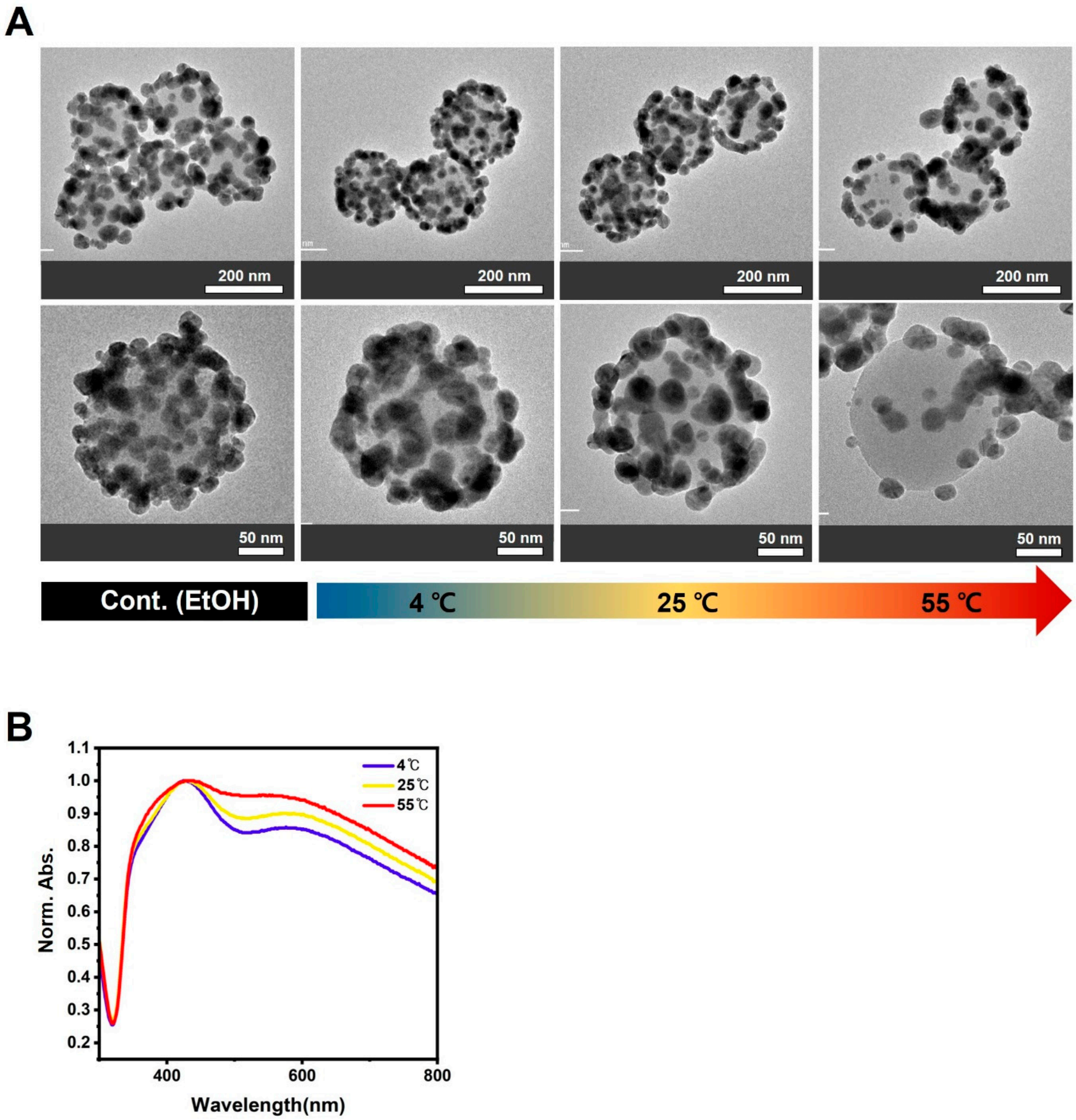

Figure S6. (A) TEM images showing structural change of SiO_2@Ag NPs depending on temperature, unlike SiO_2@Ag NPs (Cont.) stored in ethanol. **(B)** UV-Vis spectra of SiO_2@Ag NPs treated under each temperature condition (4 ℃, 25 ℃, 55 ℃) for 3 days.
